# Differences in total iron content at various altitudes of Amazonian Andes soil in Ecuador

**DOI:** 10.12688/f1000research.22411.1

**Published:** 2020-02-20

**Authors:** Benito Mendoza, Nelly Guananga, Jesus R. Melendez, Daniel A. Lowy

**Affiliations:** 1 Universidad Nacional de Chimborazo, Riobamba, Ecuador; 2 Dama Research Center limited, Kowloon, Hong Kong; 3 Escuela Superior Politécnica de Chimborazo, Riobamba, Ecuador; 4 Facultad Educación Técnica para el Desarrollo, Universidad Católica de Santiago de Guayaquil, Guayaquil, Ecuador; 5 VALOR HUNGARIAE Ltd, Budapest, Hungary

**Keywords:** Iron, total iron in soil, Hyperalic Alisol, Amazonian rainforest, Ecuador

## Abstract

Although iron is not contained by chlorophyll, it is indispensable for plants as it plays an essential role in the biosynthesis of chlorophyll. It is a component of many important plant enzyme systems, e.g. cytochrome oxidase, which is responsible for electron transport. Therefore, examining iron content of soils, particularly ionic forms of iron (Fe ^2+^ and Fe ^3+^) is important for fruit growers. In this article, we disclose the total iron content determined in soils (Hyperalic Alisol soil) at three altitudes of Amazonian rainforest in Ecuador. We examine how different altitudes impact the pH and total iron content in the selected study area. We found that total iron content significantly decreases (R2=0.966) at lower altitudes. For future studies, the authors recommend that along with Fe ion content one should determine calcium, microbial biomass, and microbial activity to better understand iron mobility and dynamics of iron uptake in the area.

## Introduction

Total iron concentration of soils mainly depends on pH ( Colombo *et al.,* 2014; Jelic *et al.,* 2010) and moisture content; and is also affected by root respiration, soil microbial activity, leaching, and erosion ( Spectrum Analytic, Inc. 2020). Given that iron deficiency is a regular problem for various crops, it is essential to determine the total iron content of soils ( Mengel *et al.,* 2001), particularly in orchards ( Simon & Szilágyi, 2003). 

In a highly cited review paper, Bünemann *et al.,* (2018) identify the most frequently used soil quality indicators under agricultural land use: organic matter, pH, available phosphate, and water storage. Soil quality evaluation should specify targeted soil threats, functions, and ecosystem services. The authors of the review recommend developing increasingly interactive assessment tools.

Recently, several studies have been undertaken on the effects on soil quality exerted by various minerals contained in the soil, such as ammonium lactate-soluble potassium and phosphorus content ( Jakab, 2020; Li *et al.,* 2020). Also investigated was the impact of various soil cultivation methods on some microbial soil properties ( Beni *et al.,* 2017; Sándor *et al.,* 2020; Sándor, 2020; Veres *et al.,* 2015).

In this article, we report the variations with altitude of the total iron content measured in intact soil in the Amazonian rainforest (in an uncultivated and uninhabited area). Considering that orchards are the most sensitive to iron deficiency, our results are aimed to support local farmers, when they select new areas for fruit plantations. An intact area was chosen as the control for soil samples, which will serve as the reference for future studies initiated in the nearby agricultural region.

## Methods

### Soil sampling

A total of 15 soil samples were collected from three altitude levels: 420, 1000, and 1600 m.a.s.l. (meters above sea level) near Tena, Ecuador, on December 10, 2019, from the upper layer (top 20 cm) of Hyperalic Alisol (Ultisols in US Soil Taxonomy) soil ( Table 1).

**Table 1.** Soil sampling points along with main physical-chemical soil properties.

№ Soil Sample	Latitude	Longitude	Altitude (m.a.s.l.)	pH (H2O)	Allophane	Moisture content (%)
1	4.628247894396525	-74.95615214109422	420	5.34	Volcanic	54.04
2	4.628247894396525	-74.95615214109422	420	5.11	Volcanic	50.48
3	4.628247894396525	-74.95615214109422	420	5.98	Volcanic	53.11
4	4.628247894396525	-74.95615214109422	420	5.01	Volcanic	55.52
5	4.628247894396525	-74.95615214109422	420	5.55	Volcanic	52.22
6	4.641091761957411	-75.02968892455102	1000	4.99	Volcanic	45.45
7	4.641091761957411	-75.02968892455102	1000	5.42	Volcanic	45.19
8	4.641091761957411	-75.02968892455102	1000	5.90	Volcanic	44.01
9	4.641091761957411	-75.02968892455102	1000	5.65	Volcanic	46.04
10	4.641091761957411	-75.02968892455102	1000	5.34	Volcanic	43.94
11	4.65093587318055	-75.09377360343935	1600	5.28	Volcanic	58.86
12	4.65093587318055	-75.09377360343935	1600	5.13	Volcanic	60.23
13	4.65093587318055	-75.09377360343935	1600	5.04	Volcanic	60.45
14	4.65093587318055	-75.09377360343935	1600	5.45	Volcanic	66.56
15	4.65093587318055	-75.09377360343935	1600	5.43	Volcanic	64.14

### Determination of soil properties

We measured pH in distilled water for soil/water ratio of 1:25 (w/w) using a glass electrode (Model Seven2Go Advanced Single-Channel Portable pH Meter, Mettler, Toledo). Soil moisture content was determined gravimetrically; drying the soil samples at 105°C for 24 h and weighing the mass loss. We measured allophane using 10.0 ± 0.5 g soil/water (1:2, w/w), soil/water plus 20. mL 1.0 M NaF, soil/water (1:2.5, w/w) + 25 mL 1.0 M NaF, soil/water (1:2.5, w/w) + 25 mL 0.50 M NaF, as described by Singla *et al.* (2018).

We determined total iron (all ionic forms) according to modified Blakemore 1981 method described in Singla *et al.*, 2018. Briefly, 50 mL of ammonium oxalate monohydrate (Spectrum Chemical) (0.20 M, pH 3) was added to 1 gram of soil sample. The mixture was shaken with a Model NB-101M Medium Orbital Shaker (N-Biotek, Inc.) in orbital mode, for 4.5 h at 150 rpms. In total 12 hours later, samples were centrifuged for 15 min at 3500 rpm (using Hermle Z400, Hermle, AG, Germany). Double filtration was performed (Whatman ^no^42 filter). A calibration curve was determined from the extracted solution (oxalate ammonium acid 0.20 M) according to Singla *et al.* (2018). The solution was measured with a Model 240Z Atomic Absorption Furnace Spectrophotometer (Agilent) at a wavelength of 392 nm and with a slit width of 0.2 nm.

### Data analysis

We applied simple linear regression (Z-test) for statistical analysis, using SPSS (version 26) to reveal possible relevant differences in pH values and total iron content at different altitudes.

## Results and discussion

Examined soil samples in the chosen area were strongly or moderately acidic, with pH values in the range from pH 4.95 ± 0.05 to pH 5.95 ± 0.05 ( Table 1). We did not find any meaningful correlation between altitude and pH values, or between pH and total iron content. Moisture content is the highest at 1600 m.a.s.l. Allophane was detected in all samples, which supports the volcanic nature of the sampling area ( Fieldes & Perrot, 1986) ( Table 1).

**Figure 1.**
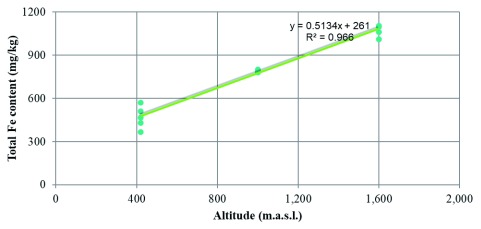
Correlation between total iron content (mg/kg) and m.a.s.l in soil samples from Amazonian Andes soil in Ecuador.

Total iron content significantly decreases (R2=0.966) at lower altitudes ( Figure 1). No significant changes in pH were found, and we can explain this by the following:

(i) vegetation at lower lying areas receive less light, so it absorbs a greater quantity of iron ions; so far, there is no relevant literature data on the effect of light intensity on the iron uptake of plants ( Borowski, 2013).
(ii) there is a greater concentration of iron-reducing bacteria in the lower lying areas, which seems to be verified by a prior study ( Fiedler *et al.,* 2007). This finding is, however, unusual, because such bacteria are typically present in sea water ( Bae *et al.,* 2001) and paddy soils ( Singla & Inubushi, 2013), rather than in Hyperalic Alisol soils.

High moisture content of the soil and organic matter accumulated on the soil surface can make air circulation difficult, hence, anaerobic conditions can develop in lower lying areas.

Our results (from 400 m.a.s.l. to 1000 m.a.s.l.) are comparable with a prior study performed in the same region ( Singla *et al.,* 2018), in which the authors report a decrease in iron content for lower laying areas. The main difference between our assessment relative Singla and colleagues’ results is that they observed a radical decrease in iron content above 1000 m.a.s.l., while we found greater iron concentrations at this altitude. Our results are comparable in magnitude to other study findings ( Fageria & Stone, 2008) carried out in South American Hyperalic Alisol soils in which high iron content was found at depths of 0–20 cm.

## Conclusions

Total iron content significantly decreases (R2=0.966) at lower altitudes. Genomics studies could detect possible iron consuming bacterial strains. For future studies, we recommend that in addition to Fe2+ and Fe3+ content one should determine calcium, microbial biomass, and microbial activity. Altogether, this approach would enable a better understanding of iron mobility and dynamics of iron uptake in the area.

## Data availability

### Underlying data

Figshare: Raw data for "Differences in total iron content at various altitudes of Amazonian Andes soil in Ecuador", https://doi.org/10.6084/m9.figshare.11833554.v2 ( Guananga, 2020).
