# Supplementary material for: Differences in total iron content at various altitudes of Amazonian Andes soil in Ecuador
Source: F1000Res. 2020 Feb 20;9:128. [Version 1] doi: 10.12688/f1000research.22411.1 (PMC7050267; doi:10.12688/f1000research.22411.1)
Supplement: Peer review history

# Peer review: Reviewer response for version 1

**DOI:** 10.5256/f1000research.24727.r60360

**Published:** 2020-02-28

**Authors:** Ankit Singla

**Affiliations:** 1 Regional Centre of Organic Farming, Ministry of Agriculture and Farmers Welfare, Government of India, Ghaziabad, Uttar Pradesh, India

***Summary of the content:***

 It should be mentioned that Kotroczo and co-workers present novel research findings considering that currently there is no iron concentration-related study published in the literature in the sampling area that examines differences in soil iron ion concentrations at different altitudional levels. As mentioned in the manuscript, this is especially useful for orchard-growers. The sampling area was well selected because of its specific location, as it is an inherit area, that is close to cultivated lands. The presented results may serve as control results for further agri- and/or horticultural studies. Also unexpected findings about the iron decrease may awaken researchers´ attention, and/or inspire a more complex research (i.e. genomic studies) in the examination area.   

**Abstract:** abstract is well written and informative.

**Keywords:** I agree with Reviewer 1 Dr. Zsolt Kotroczo that Keywords should not include those words that also could be found in the title. Please consider to replace the following keywords: *Iron, total iron, Ecuador*, providing higher possibility for search engines to find the article.

**Introduction: **One of the most challenged part of the evaluation process in case of Brief Report or Short Communications is the Introduction. Despite it is short, I believe that it consists relevant, and up-to-date research findings of the topic (mainly 2019-2020 content).

**Methods: **Methods are well written, well referenced. I agree with Reviewer 1 about it would be more fortunate to provide detailed information about the sampling area. However, it is required to clarify soil/water ratio for pH measurement was 1:25 or 1:2.5? Please check first line of determination of soil properties in the methods section.

**Results and Discussion:  **Brief and straightforward. Consists of statistical results, supporting data is available as well.

**Conclusions: **Summarizes and concludes from research findings correctly.

I confirm that I have read this submission and believe that I have an appropriate level of expertise to confirm that it is of an acceptable scientific standard.

# Peer review: Reviewer response for version 1

**DOI:** 10.5256/f1000research.24727.r60361

**Published:** 2020-02-24

**Authors:** Zsolt Kotroczo

**Affiliations:** 1 Department of Soil Science and Water Management, Szent István University, Budapest, Hungary

**Comments and suggestions**

 Authors: Benito Mendoza, Nelly Guananga, Jesus R. Melendez; Daniel A. Lowy

 Title: Differences in total iron content at various altitudes of Amazonian Andes soil in Ecuador

***Summary of the manuscript:***

 The work contains many novelties. The objectives are well founded but I miss some well-formulated hypotheses. This is very important in such well-established and well-planned research. The topic chosen increases the significance of the research.

 The manuscript is the original research paper of the authors. The subject of the manuscript is interesting and current.

 The theme of the manuscript’s scope fits the aims of the journal. Most probably the manuscript will be of great interest, contributing to the reading of the journal. It is suitable for indexing in *F1000Research *journal. The basic idea of the experiment was carried out precisely, the study provided large amount of high quality data.

 The **Abstract** is quite concise and sums up the essence of the manuscript appropriately. The abstract properly introduces the topic, summarizes the methods used and the results obtained. It is summarizes the essence of the manuscript.

**Keywords:** I suggest replacing some of the key words. The keywords that are included in the manuscript title should be replaced.

**Introduction** 

 The chapter is well structured. It fits in well with the manuscript theme, and establishes and complements the whole research topic. This chapter comprehensively supports the whole manuscript with literature. Appropriate and timely references are built in the introduction chapter. The chapter detailed and well processed.

**Methods**

 The methods chapter are appropriate and sufficiently detailed. In my opinion, soil sampling and soil analysis methods are sufficiently detailed, but the research area description is inadequate. For better identification and understanding of the results, I suggest a brief description of the sampling area.

**Results**

 The interpretation of results is generally proper. Results chapter is detailed. These chapters are well-structured and properly constructed. This chapters provide detailed and perfectly summarizes the new and novel results. It contains a number of useful experience and findings.

 The authors show the results using a graph. Evaluation of these results is appropriate and draws realistic conclusions. On the other hand, it make useful and interesting findings that may be interested.

**Conclusions**

 The chapter summarizes and well sums up the essence of the manuscript. This chapter explain and justified by the data. I think that the allegations are supported by the data and results.

 The manuscript is suitable for indexing after an above-mentioned minor revisions.

I confirm that I have read this submission and believe that I have an appropriate level of expertise to confirm that it is of an acceptable scientific standard.
